# Influence of vitreomacular interface score on treatment outcomes of anti-VEGF therapy for neovascular age-related macular degeneration

**DOI:** 10.1186/s40942-021-00342-4

**Published:** 2021-12-20

**Authors:** Manabu Miyata, Sotaro Ooto, Kenji Yamashiro, Hiroshi Tamura, Akihito Uji, Masahiro Miyake, Yuki Muraoka, Ayako Takahashi, Akitaka Tsujikawa

**Affiliations:** 1 Department of Ophthalmology and Visual Sciences, Kyoto University Graduate School of Medicine, Shogoin Kawahara Cho 54, Sakyo Ku, Kyoto, Kyoto 606-8507 Japan; 2 Department of Ophthalmology, Red Cross Otsu Hospital, Nagara 1-1-35, Otsu, Shiga 520-8501 Japan

## Abstract

**Background:**

To quantitatively evaluate the vitreomacular interface of eyes with neovascular age-related macular degeneration (AMD) and to investigate its association with the 1-year treatment outcome following intravitreal injections of aflibercept (IVA).

**Methods:**

This prospective observational case series included 59 eyes of 59 consecutive patients with treatment-naïve neovascular AMD who were treated with three monthly IVA and subsequent four bi-monthly IVA and were followed up for 1 year. We estimated posterior vitreous detachment at 1, 9, and 25 macular points within an area of 6 × 6 mm^2^ at the center of the fovea using the built-in enhanced vitreous visualization mode of swept-source optical coherence tomography. One year after the initial IVA, we classified the eyes into either wet or dry groups.

**Results:**

The wet and dry groups included 12 and 47 eyes, respectively. The resistance rate against IVA was 20.3%. The 25-point interface score was higher in the wet group than in the dry group (23.0 ± 4.3 vs. 18.6 ± 9.8, *P* = 0.03), whereas there were no significant between-group differences in the 9-point and 1-point scores (*P* = 0.21, and 0.47, respectively) or in the other studied parameters. Multivariable analysis revealed that the 25-point vitreomacular interface score was strongly correlated with subfoveal choroidal thickness (*P* = 0.02, β =  − 0.31).

**Conclusions:**

Our findings suggest that wide-ranged separation of the posterior vitreous membrane from the retina induces poor response to IVA.

## Background

Neovascular age-related macular degeneration (AMD) can lead to a rapid central vision loss because of fluid accumulation, hemorrhage, and fibrosis, as compared to dry AMD, which causes a more gradual decline in vision [1]. Although neovascular AMD represents only 10–15% of the overall prevalence of AMD, it was responsible for more than 80% of cases of severe visual loss or legal blindness before the advent of anti-vascular endothelial growth factor (VEGF) therapy [2]. Even though anti-VEGF therapy has been performed, the poor response to this treatment modality remains concerning [3].

The vitreomacular interface is reported to influence the treatment outcomes in eyes with neovascular AMD [4–9]. Vitreomacular adhesion (VMA) may be associated with AMD and is known to frequently cause vitreomacular traction (VMT) in eyes with choroidal neovascularization (CNV) [5]. VMT-affected eyes which underwent surgery have experienced a modest improvement in best-corrected visual acuity (BCVA) and decrease of retinal thickness [5]. Furthermore, remarkably firm attachment of the posterior vitreous at the macula was observed during vitrectomy in the eyes after unsuccessful photodynamic therapy [6]. VMT was also suggested to be associated with retinal pigment epithelium tear and macular hole [10, 11]. Whereas a previous study reported that VMA was associated with an inferior visual outcome after anti-VEGF therapy [7]. However, a prospective cohort study reported that VMA was not associated with visual acuity before or after intravitreal injections of ranibizumab or bevacizumab [8]. Another study showed no significant association between VMA status and visual outcomes after intravitreal injections of aflibercept (IVA) [12]. The frequency of VMA is reported to be similar among eyes with neovascular AMD, dry AMD, and no signs of AMD [9]. To date, in the literature, the relationship between VMA and AMD has remained controversial, which is different from VMT. Furthermore, the evaluations of vitreomacular interface status in the previous studies were qualitative and did not use enhanced vitreous visualization mode; therefore, the status in the entire macular area could not be assessed in detail.

In the present study, we quantitatively evaluated the vitreomacular interface status of eyes with neovascular AMD using the enhanced vitreous visualization mode of swept-source optical coherence tomography (SS-OCT) and investigated its association the 1-year treatment outcome after IVA.

## Methods

This prospective, single-center, longitudinal study was approved by the ethics committee of Kyoto University Graduate School of Medicine (Kyoto, Japan). All study protocols adhered to the tenets of the Declaration of Helsinki. All study candidates agreed to participate after providing written informed consent.

### Subjects

This study included treatment-naïve eyes affected by neovascular AMD, including typical AMD and polypoidal choroidal vasculopathy (PCV), in consecutive patients who were followed up for 1 year after receiving initial IVA (Eylea; Bayer, Basel, Switzerland) between February 2016 and April 2017 at Kyoto University Hospital. The inclusion criteria were as follows: presence of exudative or hemorrhagic features involving the macula, receiving our institution’s fixed regimen of seven IVA (three monthly injections and four bi-monthly injections) [13], a follow-up period of 1 year, and currently undergoing SS-OCT imaging. The exclusion criteria were history of intraocular surgery, including cataract surgery and vitrectomy, during the 1-year follow-up period, high myopia with an axial length (AL) of ≥ 26.00 mm, and the presence or a history of other eye diseases which may potentially induce macular edema or subretinal fluid, including diabetic retinopathy, retinal vein occlusion, or inferior posterior staphyloma. If both eyes of a patient met the eligibility criteria, the eye that was treated first was studied. Furthermore, if both eyes had received IVA on the same day, the right eye was studied.

Before IVA for neovascular AMD, all eyes underwent a comprehensive ophthalmic examination, which included BCVA measurement using a Landolt chart, AL measurement using a partial coherence interferometry, spectral domain optical coherence tomography (SD-OCT), fundus fluorescein angiography (FA) and indocyanine green angiography (ICGA), and color fundus photography. Neovascular AMD and its subtypes of typical AMD (not PCV or retinal angiomatous proliferation) and PCV were diagnosed by retinal specialists using SD-OCT and FA/ICGA images as previously reported [14]. Using SD-OCT findings, we assessed whether the eyes had retinal exudate, including subfoveal fluid, intraretinal fluid, and subretinal hemorrhage, at 1 year after the initial treatment. We assigned the eyes to either the low-response group (retinal exudate presence) or high-response group (no retinal exudate). All eyes underwent three courses of monthly injections and four subsequent courses of bi-monthly IVA. Thus, a total of seven injections should be administered during 10 months. We assessed whether the eyes had retinal exudate or no retinal exudate at 12 months after the initial treatment, which was 2 months after the final injection.

### Vitreomacular interface score

**Fig. 1:**
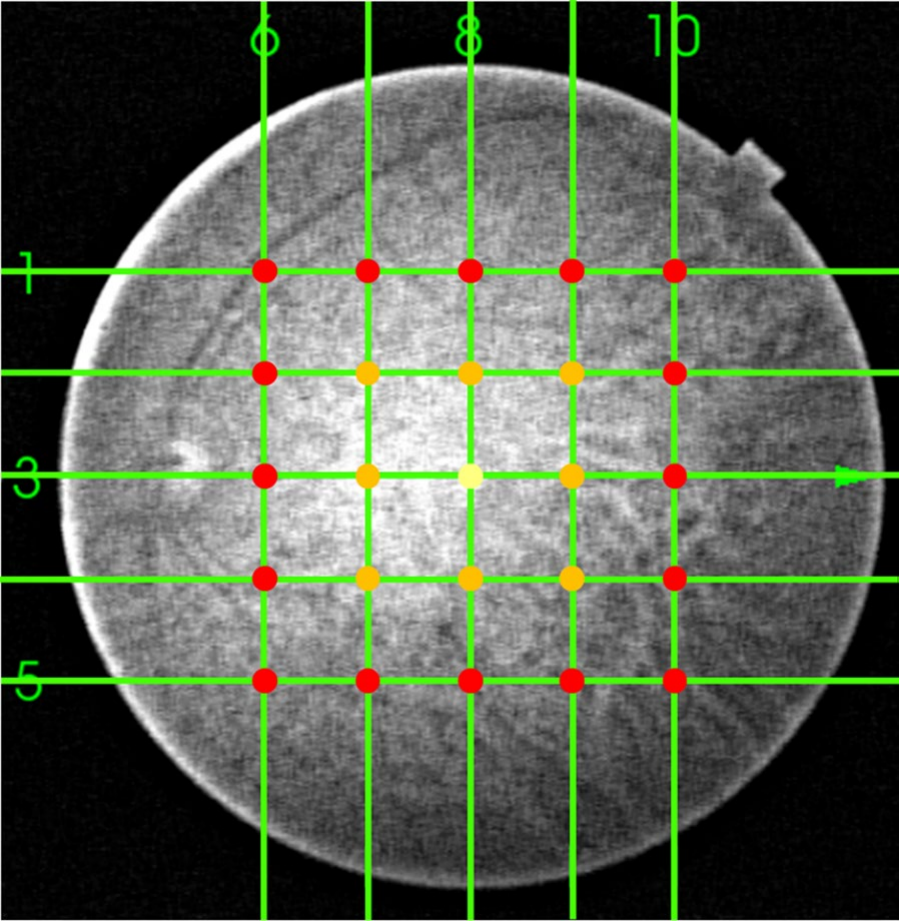
The 25 points that were analyzed to obtain vitreomacular interface scores. An investigator confirmed separation of the posterior vitreous membrane from the retina which was defined as two lines of vitreous posterior border and retinal interface using the enhanced vitreous visualization mode at 25 1.5-mm-width macular points within 6 × 6 mm^2^ areas centered the fovea. Specifically, we assessed the separation at the macular point 1 (yellow dot) at the fovea, macular points 9 (orange and yellow dots) involving vertexes and midpoint of the side of 3 × 3 mm^2^ square centered the fovea and the macular point 1, and macular points 25 (red, orange, and yellow dots) involving vertexes and points divided into quarter of the side of 6 × 6 mm^2^ square and the macular points 9. Vitreomacular interface scores were calculated by summing up the points of the separation at 1, 9, and 25 macular points

**Fig. 2:**
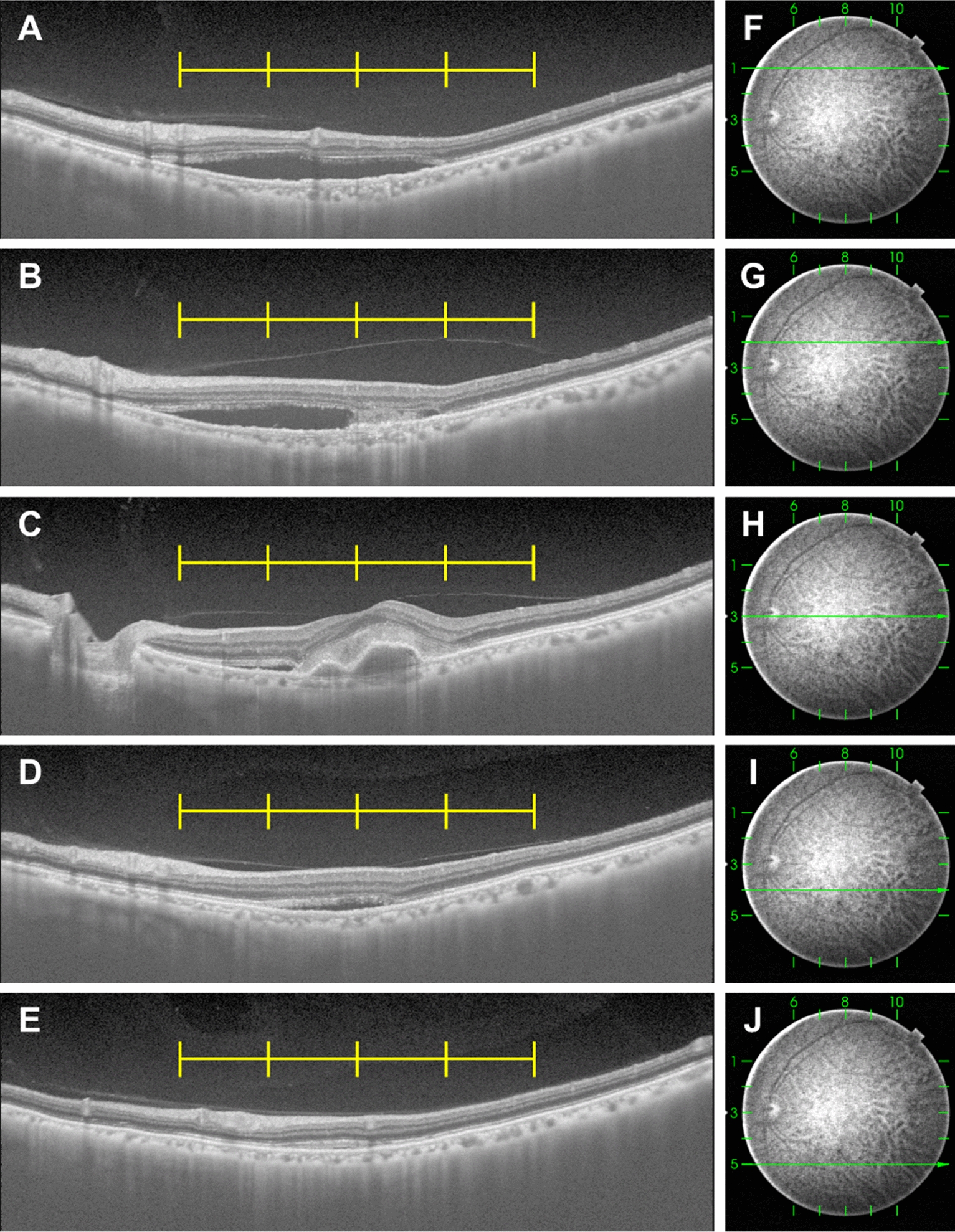
Representative images used to analyze the 25-point vitreomacular interface score. Horizontal swept- source optical coherence tomography (SS-OCT) B-scan images (**A**–**E**) and corresponding infrared images (**F**–**J**) of a left eye in a 78-year-old male patient with neovascular age-related macular degeneration, recorded before intravitreal injection of aflibercept. Yellow lines show 1.5 mm intervals centered on the fovea. **A** A horizontal SS-OCT B-scan image 3 mm above the fovea. The vitreomacular interface score was 4 by summing 1, 1, 1, 1, and 0 from the left. **B** A horizontal SS-OCT B-scan image 1.5 mm above the fovea. The vitreomacular interface score was 5 by summing 1, 1, 1, 1, and 1 from the left. **C** A horizontal SS-OCT B-scan image through the fovea. The vitreomacular interface score was 4 by summing 1, 1, 0, 1, and 1 from the left. **D** A horizontal SS-OCT B-scan image 1.5 mm beneath the fovea. The vitreomacular interface score was 3 by summing 0, 1, 0, 1, and 1 from the left. **E** A horizontal SS-OCT B-scan image 3 mm beneath the fovea. The vitreomacular interface score was 4 by summing 1, 1, 1, 1, and 0 from the left. In total, the 25-point vitreomacular interface score of this eye was 20. The 9-point score was 7, and the 1-point macular score was 0

Trained examiners performed SS-OCT (DRI OCT-1, Topcon Corp., Tokyo, Japan) evaluations after pupil dilation before IVA. During imaging, the examiners achieved pupil centration by using an internal or external fixation target. One investigator (MM) confirmed separation of the posterior vitreous membrane from the retina which was defined as two lines of vitreous posterior border and retinal interface using the enhanced vitreous visualization mode at 25 1.5-mm-width macular points within 6 × 6 mm^2^ areas centered the fovea (Fig. 1). Specifically, we assessed the separation of the posterior vitreous membrane from the retina at the macular point 1 at the fovea, macular points 9 involving vertexes and midpoint of the side of 3 × 3 mm^2^ square centered the fovea and the macular point 1, and macular points 25 involving vertexes and points divided into quarter of the side of 6 × 6 mm^2^ square and the macular points 9 (Fig. 2). Vitreomacular interface scores were calculated by summing up the points of occurrence of the separation of the posterior vitreous membrane from the retina at 1, 9, and 25 macular points. For example, 25 scores on the 25-point vitreomacular score imply that entire macular posterior vitreous detachment (PVD) has occurred within a 6 × 6 mm^2^ area. Even if complete PVD occurs and posterior vitreous border is far from the retina, the enhanced vitreous visualization mode can visualize the vitreous absence. In this case, 25-point score is 25.

Furthermore, rough vitreomacular interface status was also qualitatively analyzed because VMT has a remarkably negative effect on the treatment outcomes of anti-VEGF therapy [5, 8, 15, 16]. One investigator (MM) classified the eyes as either complete PVD, VMT, or VMA. Complete PVD was defined as PVD on every OCT scans involving the disc. VMT was defined as a steep slope of the inner macular surface or a sharp angulation and localized deformation of the retinal profile detected at the adhesion site of the hyaloid on any OCT scans [5]. VMA was defined as partial vitreomacular adhesion without VMT.

### Measurement of the central retinal thickness and subfoveal choroidal thickness

Central retinal thickness was defined as the distance between the inner surface of the internal limiting membrane and the inner surface of the retinal pigment epithelium beneath the fovea. Subfoveal choroidal thickness was defined as the distance between the Bruch’s membrane and the interface of the sclera. Before treatment, one investigator (MM) measured these thicknesses on SS-OCT images using the built-in software.

### Genotype

Genomic DNA was extracted from peripheral blood samples using a DNA extraction kit (QuickGene-610 L; Fujifilm, Minato, Tokyo, Japan). Genes encoding for the ARMS2 A69S rs10490924 and complement factor H I62V rs800292 were genotyped by the TaqMan single-nucleotide polymorphism assay method using the ABI PRISM 7700 system (Applied Biosystems, Foster City, CA, USA), as previously reported [17].

### Statistical analysis

All data were presented as means ± standard deviations where applicable. For statistical analyses, all BCVA values were converted to logarithm of the minimum angle of resolution (logMAR) units. Comparative analyses were performed using the *t*-test, chi-square test, or chis-square trend test where applicable. Correlation analyses between the 25-point vitreomacular interface score and the other studied parameters were performed using the Spearman’s rank correlation coefficient analysis. Multiple stepwise regression analyses were performed using the 25-point vitreomacular interface score as a dependent variable and the other parameters (except vitreous-associated parameters) with *P-*values of < 0.10 by Spearman’s correlation coefficients as independent variables. A *P*-value of < 0.05 was considered statistically significant. All statistical analyses were performed using the SPSS version 21 software (IBM Corp., Armonk, NY, USA).

## Results

In total, 59 eyes of 59 consecutive patients (74.3 ± 7.9 years, 18 females) were included (Table 1). BCVA improved 1 year after IVA (*P* = 0.006, baseline and 1-year logMAR BCVA; 0.28 ± 0.32, 0.18 ± 0.35, respectively). Thirty and 29 eyes were affected with typical AMD and PCV, respectively. Homozygous risk allele at ARMS2 A69S rs10490924 and complement factor H I62V rs800292 was observed in 20 and 21 patients, respectively. Based on the estimation of qualitative vitreomacular interface status, VMA and complete PVD were observed in 16 (27.1%) and 43 patients (72.9%), respectively. No VMT was observed in the eyes.

**Table 1:** Comparison between the wet and dry groups

	All eyes	Low-response group	High-response group	*P*-value
Eyes, n (patients, n)	59 (59)	12 (12)	47 (47)	
Age, years (range)	74.3 ± 7.9 (50–88)	77.0 ± 5.9 (66–85)	73.7 ± 8.2 (50–88)	0.19
Sex (M/F), n	41/18	7/5	34/13	0.48^†^
Subtype (tAMD/PCV)	30/29	6/6	24/23	1.00^†^
Axial length, mm	23.74 ± 0.91	23.61 ± 1.05	23.77 ± 0.89	0.59
LogMAR BCVA				
Baseline	0.28 ± 0.32	0.31 ± 0.37	0.27 ± 0.31	0.69
1 year after treatment	0.18 ± 0.35	0.21 ± 0.24	0.17 ± 0.38	0.74
Change during 1 year	− 0.10 ± 0.27	− 0.10 ± 0.27	− 0.10 ± 0.27	0.96
Central retinal thickness at baseline, μm	280.4 ± 113.6	306.8 ± 118.8	273.7 ± 112.5	0.37
Subfoveal choroidal thickness at baseline, μm	231.8 ± 111.3	209.9 ± 90.4	237.4 ± 116.2	0.45
Genotype, n				
*ARMS2* A69S (GG/GT/TT)	11/26/20^a^	1/6/4^b^	10/20/16^c^	0.60^‡^
*CFH* I62V (AA/AG/GG)	6/30/21^d^	1/5/5^e^	5/25/16^f^	0.54^‡^
Vitreomacular interface scores at baseline				
25 points	19.5 ± 9.1	23.0 ± 4.3	18.6 ± 9.8	0.03*
9 points	6.5 ± 3.7	7.6 ± 3.0	6.3 ± 3.8	0.21
1 point	0.7 ± 0.5	0.8 ± 0.5	0.6 ± 0.5	0.47
Qualitative vitreomacular interface status at baseline (VMA/VMT/complete PVD), n	16/0/43	1/0/11	15/0/32	0.10^‡^
Qualitative vitreomacular interface status at baseline (VMA/complete PVD), n	16/43	1/11	15/32	0.10^†^

Data are presented as means ± standard deviations where applicable

Low-response group: subretinal and/or intraretinal fluid observed on optical coherence tomography 1 year after the initial treatment

High-response group: no subretinal or intraretinal fluid observed on optical coherence tomography 1 year after the initial treatment

tAMD: typical age-related macular degeneration; PCV: polypoidal choroidal vasculopathy; logMAR BCVA: logarithm of the minimal angle of resolution best-corrected visual acuity; *ARMS2*: age-related maculopathy susceptibility protein 2; *CFH*: complement factor H; PVD: posterior vitreous detachment; VMA: vitreomacular adhesion without traction; VMT: vitreomacular traction

In *ARMS2* A69S, GG, GT, and TT represent non-risk-homo, hetero, and risk-homo, respectively

In *CFH* I62V, AA, AG, and GG represent non-risk-homo, hetero, and risk-homo, respectively

In measurements indicated by ^a^, ^b^, ^c^, ^d^, ^e^, and ^f^, data are missing for two, one, one, two, one, and one patients, respectively

^†^Chi-square test

^‡^Chi-square trend test; the remaining: *t*-test

*Statistically significant (*P* < 0.05)

Twelve eyes (20.3%) and 47 eyes (79.7%) were assigned to the low-response and high-response groups, respectively (Table 1). Thus, the poor responder rate to seven fixed regimens of IVA was 20.3%. The 25-point vitreomacular interface score was higher in the low-response group than in the high-response group (23.0 ± 4.3 vs. 18.6 ± 9.8, *P* = 0.03), whereas there was no significant difference in the 9-point and 1-point scores between the two groups (*P* = 0.21, and 0.47, respectively). Furthermore, there was no difference in qualitative vitreomacular interface status between the two groups. There were no significant differences in the other baseline parameters, such as age, sex, subtype, AL, logMAR BCVA, central retinal thickness, subfoveal choroidal thickness, or genotype. Change of logMAR BCVA during 1 year was also not significantly different between the two groups (*P* = 0.96).

Univariable analysis revealed that the 25-point vitreomacular interface score was significantly correlated with age (*P* = 0.04, r = 0.27), baseline and 1-year BCVA (*P* = 0.02, r = 0.30; *P* = 0.04, r = 0.28), subfoveal choroidal thickness (*P* = 0.01, r =  − 0.32), and PVD status (*P* < 0.001, r = 0.86) (Table 2). Multivariable analysis revealed that the 25-point vitreomacular interface score was correlated with subfoveal choroidal thickness (*P* = 0.02, β =  − 0.31) when vitreous-associated parameters were excluded.

**Table 2:** Correlation between 25-point vitreomacular interface score and other studied Parameters

Clinical parameters	Univariable analysis		Multivariable analysis	
	*P*	r	*P*	β
Age	0.04*	0.27	0.48	0.10
Sex (1, male; 2, female)	0.58	0.07		
Subtype (1, tAMD; 2, PCV)	0.46	0.10		
Axial length	0.43	0.11		
LogMAR BCVA				
Baseline	0.02*	0.30	0.11	0.21
1-year follow-up	0.04*	0.28	0.24	0.16
Central retinal thickness at baseline	0.51	− 0.09		
Subfoveal choroidal thickness at baseline	0.01*	− 0.32	0.02*	− 0.31
Genotype				
*ARMS2* A69S (0, GG; 1, GT; 2, TT)	0.06^a^	0.25	0.51	0.09
*CFH* I62V (0, AA; 1, AG; 2, GG)	0.06^b^	− 0.25	0.20	− 0.17

Data are presented as means ± standard deviations where applicable

tAMD, typical age-related macular degeneration; PCV, polypoidal choroidal vasculopathy; logMAR BCVA, logarithm of the minimal angle of resolution best-corrected visual acuity; *ARMS2*, age-related maculopathy susceptibility protein 2; *CFH*, complement factor H

In measurements indicated by ^a^ and ^b^, data are missing for two and two patients, respectively

*Statistically significant (*P* < 0.05)

Comparing studied parameters between eyes with complete PVD (43/59) and eyes without it (16/59) based on qualitative estimation of vitreomacular interface status, the eyes with complete PVD were older (*P* = 0.03) and had thinner choroid (*P* = 0.003) (Table 3). However, there were no significant differences in logMAR BCVA at baseline, logMAR BCVA 1 year after treatment, and change of logMAR BCVA between the two groups. The rate of wet status was higher in the eyes with complete PVD than in those without it; however, the difference was not significant (*P* = 0.10).

**Table 3:** Comparison between complete PVD and the other

	Complete PVD	The other	*P*-value
Eyes, n (patients, n)	43 (43)	16 (16)	
Age, years	75.7 ± 7.3	70.7 ± 8.3	0.03*
Sex (M/F), n	29/14	12/4	0.58^†^
Subtype (tAMD/PCV)	20/23	10/6	0.28^†^
Axial length, mm	23.81 ± 0.88	23.54 ± 1.01	0.31
LogMAR BCVA			
Baseline	0.32 ± 0.34	0.16 ± 0.25	0.10
1 year after treatment	0.22 ± 0.38	0.06 ± 0.24	0.11
Change during 1 year	− 0.08 ± 0.25	− 0.15 ± 0.31	0.37
Central retinal thickness at baseline, μm	275.4 ± 115.1	293.9 ± 112.0	0.58
Subfoveal choroidal thickness at baseline, μm	206.2 ± 93.9	300.5 ± 127.6	0.003*
1-year retinal exudate status (wet/dry)	11/32	1/15	0.10^†^
Genotype, n			
*ARMS2* A69S (GG/GT/TT)	8/19/14^a^	3/7/6	0.14^‡^
*CFH* I62V (AA/AG/GG)	4/20/17^b^	2/10/4	0.42^‡^

Data are presented as means ± standard deviations where applicable

PVD, posterior vitreous detachment; tAMD, typical age-related macular degeneration; PCV, polypoidal choroidal vasculopathy; logMAR BCVA, logarithm of the minimal angle of resolution best-corrected visual acuity; *ARMS2*, age-related maculopathy susceptibility protein 2; and *CFH*, complement factor H

In *ARMS2* A69S, GG, GT, and TT represent non-risk-homo, hetero, and risk-homo, respectively

In *CFH* I62V, AA, AG, and GG represent non-risk-homo, hetero, and risk-homo, respectively

In measurements indicated by ^a^ and ^b^ data are missing for two and two patients, respectively

^†^Chi-square test

^‡^Chi-square trend test; the remaining: *t*-test

*Statistically significant (*P* < 0.05)

## Discussion

In the present study, the enhanced vitreous visualization mode of SS-OCT enabled us to determine the separation of the posterior vitreous membrane from the retina at each macular point. The 25-point vitreomacular score was significantly higher in the low-response group than in the high-response group, which indicates that AMD-affected eyes with high 25-point scores have a poor response to IVA. However, the 1-point and 9-point vitreomacular scores and qualitative vitreomacular interface status were not different between the two groups. Vitreomacular interface status except for VMT might not play so important role in treatment on AMD. Furthermore, there was no difference in 1-year BCVA change between the two groups. Taken together, eyes with wide-ranged separation of the posterior vitreous membrane from the retina at baseline appear to be more likely to have active disease 1 year after initial IVA, although there was no impact on 1-year BCVA change.

Our fine estimation of vitreomacular interface status suggested that high 25-point score induces poor response to anti-VEGF therapy 1 year after the initial treatment (*P* = 0.03), which implies that broader VMA affects better response to anti-VEGF therapy. However, there was no difference in BCVA between high- and low-response groups, which agrees with previous reports showed no association of VMA based on qualitative estimation and BCVA during the 6-month to 2-year observation period [8, 12]. Unexpectedly, FLUID study reported the non-inferiority of BCVA in tolerated some subretinal fluid (SRF) after anti-VEGF therapy in neovascular AMD compared to complete resolution of SRF 24 months after the treatment [18]. However, BCVA decreases in chronic central serous chorioretinopathy, in which SRF maintains for long term, during 10-year follow up period [19]. Further long-term study of SRF in neovascular AMD might show the association between response to IVA and BCVA and significance of our findings.

Rough qualitative estimation suggests that the baseline complete PVD tended to induce poor response (*P* = 0.10). The vitreous gel maintains the concentration of the anti-VEGF agent, which indicates that PVD may decrease the amount of vitreous gel and subsequently cause early clearance of the therapeutic agent. However, PVD is known to increase vitreal O_2_ levels [20] and decrease the aqueous concentration of VEGF [21]. Thus, PVD plays a role in decreasing both concentration of anti-VEGF agent and its targeted VEGF. It is unclear which effect on activity of AMD was higher. Considering our results, the effect of the decreasing concentration of anti-VEGF agent by PVD might be slightly higher. However, a previous report showed that the 1-year numbers of additional anti-VEGF therapy on PRN regimen was 4.9 in PVD and 5.3 in VMA, which are similar [22]. Furthermore, when comparing studied parameters between eyes with complete PVD and eyes without it, there were no significant differences in baseline and 1-year logMAR BCVA. A previous report using ultrasonography showed the similar results [23]. Taken together, although VMT obviously affects treatment outcome of anti-VEGF therapy in AMD [5, 15], PVD and VMA has remained controversial. Our results suggested that PVD negatively affects to decrease activity of AMD by anti-VEGF therapy.

Multivariable correlation analysis showed that 25-point vitreomacular interface scores were negatively associated with subfoveal baseline choroidal thickness. This result is consistent with analysis between complete PVD and the other (all the VMA in the present study). Thus, subfoveal choroid with complete PVD, high 25-point score, was significantly thinner (complete PVD 206.2 ± 93.9 μm, others 300.5 ± 127.6). Pachychoroid, which is recently proposed, is characterized by thick choroid [24]. A previous study showed that pachychoroid neovasculopathy had a similar effect of anti-VEGF therapy on visual outcome; however, pachychoroid neovasculopathy required lesser number of treatments, which means a higher response, compared to neovascular AMD [25]. Pachychoroid might indirectly affect response to anti-VEGF therapy through the vitreomacular interface score.

A previous large cohort study showed that VMT and VMA were observed in 1.8% and 11.0% of AMD-affected eyes, respectively [8]. Therefore, VMT was a rare occurrence in AMD. In the present study, VMT was not observed and VMA was observed in 20.0% of eyes, which is similar to the prevalence of the previous study. Because VMT obviously negatively affect treatment outcome of anti-VEGF for neovascular AMD [5, 8, 15, 16], VMT should be excluded in estimating effects of fine vitreomacular interface, if it is present.

The present study had some limitations. First, the sample size was small. Twenty-five patients met the exclusion criteria and dropped out, after which 59 patients were analyzed. Further studies with larger sample sizes are necessary to support our findings. Second, the physiological mechanism responsible for our findings remains unclear. Further studies including subjects of different races are necessary to validate our results.

In conclusion, our 25-point vitreomacular interface score obtained through fine analysis using the enhanced vitreous visualization mode of SS-OCT suggests that wide-ranged separation of the posterior vitreous membrane from the retina at baseline induces poor response to IVA.

## Data Availability

All data generated or analyzed during this study are included in this published article.

## Abbreviations

AMD: Age-related macular degeneration
AL: Axial length
BCVA: Best-corrected visual acuity
CNV: Choroidal neovascularization
FA: Fluorescein angiography
ICGA: Indocyanine green angiography
IVA: Intravitreal injections of aflibercept
logMAR: Logarithm of the minimum angle of resolution
PCV: Polypoidal choroidal vasculopathy
PVD: Posterior vitreous detachment
SD-OCT: Spectral domain optical coherence tomography
SS-OCT: Swept-source optical coherence tomography
VEGF: Vascular endothelial growth factor
VMA: Vitreomacular adhesion
VMT: Vitreomacular traction
